# MASLD beyond the liver: decoding the gut–genetic–metabolic nexus

**DOI:** 10.3389/fgstr.2026.1824548

**Published:** 2026-09-22

**Authors:** Philippe Attieh, Maria Nassif, Mark Maghzal, Anthony Bedran, Jane Kassas, Mahmoud Othman, Francois Sahyoun, Frederic Harb, Sami Azar, Hilda E. Ghadieh

**Affiliations:** 1 Department of General Surgery, Faculty of Medicine and Medical Sciences, University of Balamand, Al-Koura, Tripoli, Lebanon; 2 Department of Biomedical Sciences, Faculty of Medicine and Medical Sciences, University of Balamand, Al-Koura, Tripoli, Lebanon; 3 Department of Gastroenterology, Faculty of Medicine and Medical Sciences, University of Balamand, Al-Koura, Tripoli, Lebanon; 4 Department of Internal Medicine, Faculty of Medicine and Medical Sciences, University of Balamand, Al-Koura, Tripoli, Lebanon

**Keywords:** gut-liver axis, hepatocellular carcinoma, metabolic dysfunction-associated steatotic liver disease (MASLD), metabolic syndrome, microbiome dysbiosis

## Abstract

Metabolic dysfunction–associated steatotic liver disease (MASLD), formerly non-alcoholic fatty liver disease, is the most prevalent chronic liver disorder globally and is tightly linked to obesity, diabetes, dyslipidemia, and metabolic syndrome. MASLD spans a continuum from simple steatosis to metabolic dysfunction–associated steatohepatitis, fibrosis, cirrhosis, and hepatocellular carcinoma. Its pathogenesis is driven by complex interactions among host genetics, metabolic stress, dietary factors, gut microbiota dysbiosis, immune dysregulation, and oxidative injury. The gut–liver axis plays a central role, with microbial metabolites and altered intestinal permeability driving hepatic inflammation and fibrogenesis. Genetic variants, including PNPLA3, TM6SF2, and MBOAT7, modulate disease susceptibility and metabolic pathways. Emerging therapies target interconnected mechanisms through lifestyle interventions, microbiome-directed strategies, bile acid signaling, incretin-based treatments, and antifibrotic approaches. Advances in omics technologies and biomarkers support precision medicine frameworks. Despite major advances in mechanistic understanding, resmetirom has recently become the first FDA-approved therapy for adults with non-cirrhotic MASH and moderate-to-advanced fibrosis. Nevertheless, effective treatment options remain limited, emphasizing the need for additional mechanism-based and personalized therapeutic strategies.

## 1 Introduction

Nonalcoholic fatty liver disease (NAFLD) is a metabolic liver disorder marked by excessive triglyceride (TG) accumulation in hepatocytes (1). It encompasses a spectrum of conditions, from SFL and non-alcoholic fatty liver (NAFL) to non-alcoholic steatohepatitis (NASH), cirrhosis, and even hepatocellular carcinoma (HCC) in advanced stages (2). With its rising incidence at younger ages and its association with cancer risk, NAFLD has become a growing health concern (3). The traditional “two-hit hypothesis” suggested that progression from steatosis to NASH required a secondary trigger, but this concept has evolved with recognition of multiple contributing factors, including genetics, diet, environmental exposures, alcohol, smoking, insulin resistance, and even oral health issues such as periodontitis (4). Reflecting this broader understanding, the terminology has shifted, with NAFLD now referred to as metabolic dysfunction-associated steatotic liver disease (MASLD), and NASH as metabolic dysfunction-associated steatohepatitis (MASH) (5). MASLD frequently coexists with metabolic syndrome and involves diverse pathogenic pathways, leading to heterogeneity in clinical presentation. Current models emphasize hepatic metabolic overload, toxic lipid accumulation, cellular stress, and hepatocyte injury (6). Despite these advances, therapeutic options remain limited. The recent FDA approval of the thyroid hormone receptor-β agonist resmetirom for adults with non-cirrhotic MASH and stage F2–F3 fibrosis represents an important milestone (7); however, additional therapies are still needed to address the heterogeneous spectrum of MASLD and advanced disease stages. Rather than considering these mechanisms as isolated processes, this review adopts the concept of a gut–genetic–metabolic nexus, in which inherited genetic susceptibility establishes the biological background upon which metabolic dysfunction develops, while gut microbiota composition and function dynamically modulate disease initiation, progression, and therapeutic responsiveness. Throughout this review, we examine how these interconnected systems collectively determine MASLD heterogeneity, emphasizing that disease progression reflects continuous bidirectional interactions rather than linear pathogenic events. By integrating current evidence, we highlight the role of microbiota, metabolic dysregulation, and immune signaling in disease progression, while outlining promising diagnostic biomarkers and therapeutic strategies to guide future research and clinical management.

## 2 Pathophysiology of MASLD and the gut microbiome

### 2.1 Introduction to the gut–liver axis

The gut-liver axis is the anatomic and functional connection between the gut and liver via the portal vein and biliary tract. In MASLD, dysbiosis, increased intestinal permeability, and altered microbial metabolite production disrupt this axis, promoting steatosis, inflammation, and fibrosis (8). Thus, the gut microbiome is not only an association but also a central contributor to MASLD pathophysiology.

### 2.2 Gut microbiome alterations in MASLD

Studies consistently report distinct dysbiosis in MASLD. Hallmarks include firstly, increased pathogenic taxa like ethanol- and endotoxin-producing bacteria. Secondly, depletion of beneficial commensals reduced butyrate-producing bacteria such as Faecalibacterium, and lastly, functional reprogramming or altered microbial capacity for bile acid transformation, choline metabolism, and tryptophan catabolism.

These microbial alterations correlate with hepatic fat accumulation, insulin resistance, and inflammatory activity, supporting a mechanistic link between gut microbiome dysfunction and liver injury. Although experimental studies, including germ-free and fecal microbiota transplantation models, provide evidence for a contributory causal role, most available human studies remain observational and demonstrate associations rather than definitive causality (8, 9).

### 2.3 Mechanisms of barrier injury

Dietary factors (high fat, high sugar), dysbiosis, and reduced short-chain fatty acids (SCFA) availability compromise the integrity of the intestinal barrier. When tight-junction integrity is impaired, it leads to a “leaky gut” which in turn has consequences for the liver (10).

Barrier dysfunction allows translocation of microbial products: Lipopolysaccharide (LPS) activate Toll-like receptor 4 (TLR-4) on Kupffer cells which in turn upregulate Necrosis factor kappa B (NF-κB) and cytokine production such as tumor necrosis factor alpha (TNF-α) andinterleukin-1β (IL-1β)) generating a fibrogenic response and interfering with lipid and glucose homeostasis. Furthermore, flagellin and peptidoglycans stimulate innate immune pathways and small-intestinal bacterial overgrowth (SIBO) further increases endotoxin flux. The epithelial barrier hypothesis positions leaky gut as the first hit that triggers hepatic immune activation and sets the stage for MASH (8).

### 2.4 Microbial metabolites driving MASLD progression

The gut microbiota contributes to MASLD, not only through the disruption of intestinal barrier’s integrity, but also via the excretion of bioactive substances that may alter the liver’s signaling pathways (**Table 1**).

**Table 1:** Microbial metabolites driving MASLD progression.

Metabolite	Source/Mechanism	Effect on Liver	Reference
Bile acids	Dysbiosis alters bile acid composition; affects FXR and TGR5 signaling	Impaired metabolism, toxic secondary bile acids → steatosis, inflammation, fibrosis, hepatocyte injury	(8)
SCFAs	Butyrate from microbial fermentation of dietary fibers	Maintains gut barrier, regulates lipid metabolism; deficiency → leaky gut, insulin resistance, steatosis	(8)
IPA, IAA	Produced from tryptophan metabolism by tryptophanase-expressing bacteria	Activate AHR and PXR → detoxification, protective signaling; supplementation improves steatosis	(11)
Choline & TMAO	Gut bacteria convert choline → trimethylamine → TMAO by host	Reduces hepatic phosphatidylcholine, impairs VLDL export → insulin resistance, worsens MASLD	(8)
Endogenous ethanol & phenylacetic acid	Microbial fermentation products (ethanol, phenylacetic acid)	Ethanol → oxidative stress & peroxidation; phenylacetic acid → stimulates lipogenesis	(8)
Other co-metabolites	Sphingolipids, phenolics, secondary bile acid derivatives	Promote inflammatory processes and hepatocyte injury	(12)

↑, increased or upregulated; ↓, decreased or downregulated; →, leads to, promotes, or results in the subsequent effect/pathway.

#### 2.4.1 Bile acids

When it comes to bile acids, disruption of the gut microbiome, known as dysbiosis, alters bile acid composition and signaling through farnesoid X receptor (FXR) and takeda G protein- coupled receptor 5 (TGR5), leading to an impaired regulation of hepatic metabolism, including the processes of energy consumption, weight loss and glucose homeostasis; resulting in increased hepatic bile acid synthesis especially secondary bile acids that are known to be poorly water soluble and toxic to cells, therefore aggravating hepatic steatosis, inflammation, fibrosis and hepatocyte injury (8).

#### 2.4.2 SCFA

Butyrate is a SCFA, produced by microbial fermentation of dietary fibers in the lower intestinal tract, known to feed the cells lining the colon and to promote a healthy gut barrier. A reduction in butyrate production contributes to a leaky gut also known as intestinal hyperpermeability as well as increases insulin resistance. SCFAs regulate lipid metabolism via G protein coupled receptor on the surface of hepatocytes and their deficiency largely worsens steatosis (8).

#### 2.4.3 Indole derivatives

Indole is produced by bacteria that express tryptophanase. These bacteria metabolize tryptophan into indole and subsequently into indole 3-propionic acid (IPA) and indole acetic acid (IAA) that are known to activate aryl hydrocarbon receptor (AHR) and pregnane X receptor (PXR) that are both transcription factors in the cell nucleus to protect cells from toxic insults, they induce the expression of enzymes and drug transporters that biotransform xenobiotics and endobiotics and eliminate toxic metabolites. Supplementation with IPA/IAA was shown to improve hepatic steatosis in MASLD patients (11).

#### 2.4.4 Choline and trimethylamine N oxide

Gut microorganisms can anaerobically convert choline to trimethylamine, which is further metabolized to TMAO by the host leading to a reduction of hepatic phosphatidylcholine needed for the export of very low density lipoproteins (VLDL) that carry TG to peripheral tissues. Elevated TMAO links dysbiosis with insulin resistance and worsens MASLD course (8).

#### 2.4.5 Endogenous ethanol and phenylacetic acid

Ethanol is a major microbial metabolite that is produced in the intestine of nearly all individuals, increased ethanol production is associated with a wide array of metabolic dysfunction including elevated oxidative stress and peroxidation, while microbial phenylacetic acid stimulates hepatic lipogenesis which consistent of converting carbohydrates into fatty acids for long term energy storage (8).

Other co metabolites: sphingolipids, phenolics and secondary bile acid derivatives further contribute to intracellular inflammatory processes, expanding the range of microbiota-derived harmful signals (12).

### 2.5 Oxidative stress and exogenous amplifiers

Oxidative stress is the bridge linking microbial signals with hepatic injury. Reactive oxygen species induced by microbial ethanol and LPS drive mitochondrial dysfunction, lipid peroxidation, and hepatocyte apoptosis (4) while nicotine exposure worsens dysbiosis, barrier disruption, and redox imbalance, therefore, amplifying MASLD severity (13). This oxidative–microbial axis is a critical determinant of disease progression.

### 2.6 Adipose tissue-liver crosstalk

Adipose tissue dysfunction is increasingly recognized as a fundamental upstream driver of MASLD that acts in concert with gut-derived mechanisms. In obesity, hypertrophic adipocytes exhibit impaired lipid storage capacity, resulting in increased release of free fatty acids into the portal and systemic circulation. This excess lipid flux promotes hepatic triglyceride accumulation, lipotoxicity, mitochondrial dysfunction, and oxidative stress. Simultaneously, inflamed adipose tissue secretes increased levels of pro-inflammatory adipokines and cytokines, including tumor necrosis factor-α, interleukin-6, and leptin, while reducing protective adiponectin concentrations (4, 14, 15). These alterations exacerbate hepatic insulin resistance, activate Kupffer cells and hepatic stellate cells, and promote fibrogenesis. Importantly, gut-derived endotoxins such as lipopolysaccharide further amplify adipose tissue inflammation through Toll-like receptor signaling, establishing a bidirectional adipose–gut–liver axis that perpetuates metabolic dysfunction. Thus, adipose tissue should be regarded not merely as an energy storage organ but as a central endocrine and immunometabolic regulator that interacts closely with the gut microbiome to influence MASLD progression (4, 14, 15).

### 2.7 Endocrine crosstalk: apelin as a mediator

Adipokines are also implicated in the pathophysiology of MASLD. Apelin, an adipokine, is elevated in MASLD, and was shown to be correlated with advanced stages of fibrosis and increased HCC risk. In hepatic stellate cells, apelin activates pro-fibrotic pathways leading to collagen deposition and liver fibrosis. A correlation between Apelin concentration and pro-inflammatory cytokines like IL-6 and TNF was established, suggesting a potential effect in inflammatory processes. Apelin, therefore, can be a potential marker for disease severity and progression (16).

Although the gut microbiome represents a key mechanistic component of MASLD, current evidence indicates that it functions primarily as a disease modifier rather than the principal initiating factor in most patients. Obesity, insulin resistance, adipose tissue dysfunction, and inherited genetic variants remain the dominant determinants of disease susceptibility and hepatic lipid accumulation (4, 17). Gut microbial dysbiosis amplifies these pathogenic processes by promoting intestinal permeability, endotoxemia, altered bile acid signaling, and immune activation, thereby accelerating progression from simple steatosis toward steatohepatitis and fibrosis. Importantly, the microbiome remains an attractive therapeutic target because, unlike host genetics, it is potentially modifiable through dietary interventions, pharmacologic therapies, and microbiome-directed approaches (4, 17).

In summary, MASLD pathophysiology can be conceptualized in five steps. Firstly, metabolic dysfunction and diet lead to gut dysbiosis. This dysbiosis, impairs metabolite balance (reduced SCFAs/indoles; increased ethanol/TMAO) which in turn causes barrier breakdown and portal influx of LPS and microbial toxins. Hepatic signaling (FXR/TGR5, AHR/PXR, TLR4/NLRP3) is activated and induces steatosis, inflammation, cell injury. Then finally, amplifiers like oxidative stress, nicotine and apelin dysregulation aggravate fibrosis and progression to advanced disease. Understanding these mechanisms could provide several potential intervention points. These include barrier restoration, bile acid modulation, targeted reduction of harmful metabolites (ethanol, TMAO) through microbial modulation, oxidative stress reduction via antioxidants and lifestyle changes and adipokine monitoring (apelin) as a biomarker of fibrosis risk.

## 3 Host–microbiome interactions: genetics, epigenetics, hormones, and signaling

Building upon the gut–liver axis described previously, this section focuses on the second component of the proposed conceptual framework by examining how host genetics and epigenetic regulation interact with microbial and metabolic pathways to influence disease susceptibility and progression.

MASLD represents a global concern as more than 25% of adults are affected by this hepatic consequence of systemic metabolic dysregulation. In fact, MASLD can range from a simple steatosis to full metabolic steatohepatitis, fibrosis, or even HCC (**Figure 1**). Its broad pathogenesis makes it even more challenging as genetic, hormonal, microbial and dietary components play a major role in the disease’s progression (**Table 2**). The gut-liver axis, where gut-derived metabolites and microbial components, is an important factor influencing hepatic inflammation, immune tone and lipid metabolism (18, 19).

**Figure 1:**
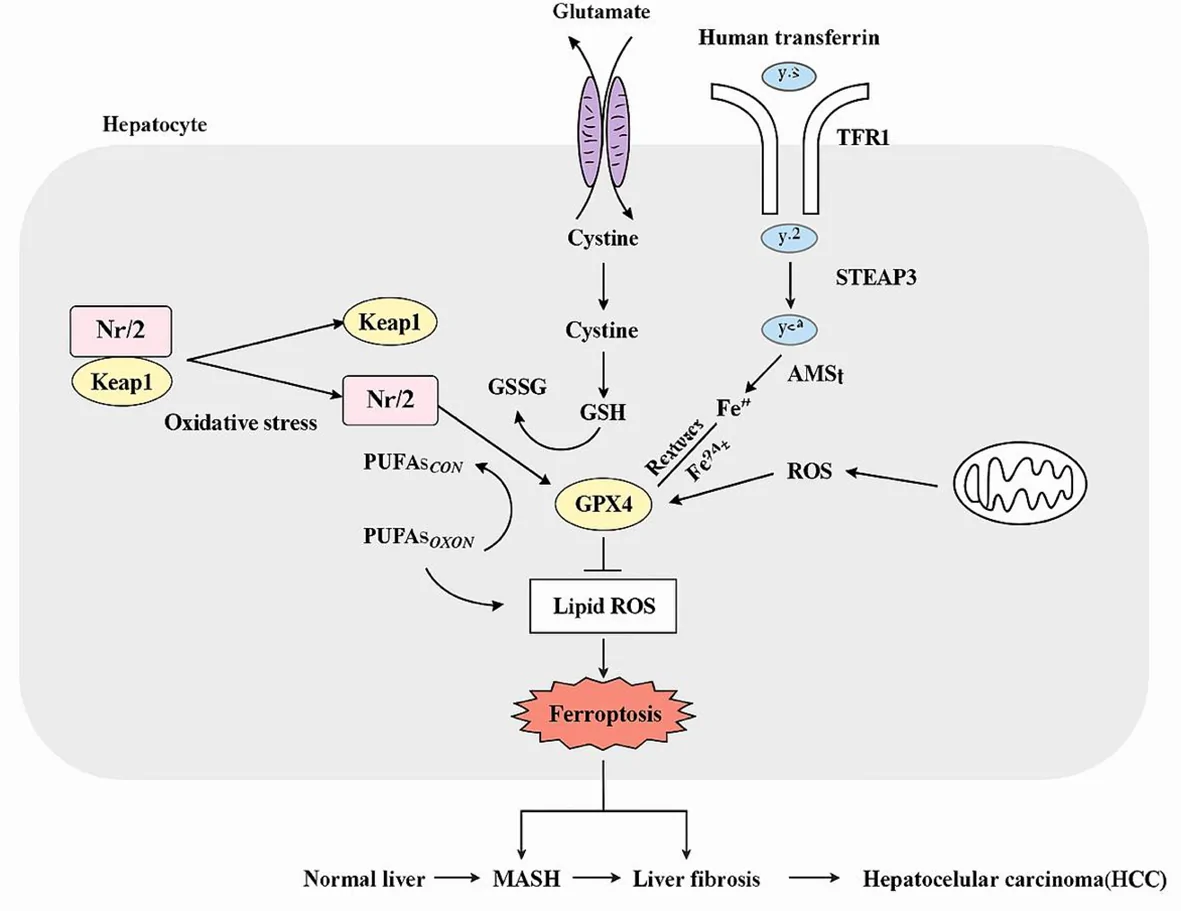
MASLD pathogenesis in terms of host–microbiome interactions: genetics, epigenetics, hormones, and signaling. This schematic illustrates the multifactorial processes involved in MASLD development. Metabolic dysfunction, insulin resistance, and genetic susceptibility promote hepatic lipid accumulation and lipotoxicity. Gut microbiota dysbiosis increases intestinal permeability, facilitating the translocation of microbial products such as lipopolysaccharide (LPS) into the portal circulation, thereby activating hepatic innate immune responses through Toll-like receptor signaling. Oxidative stress, mitochondrial dysfunction, inflammatory cytokine production, and hepatic stellate cell activation subsequently promote fibrosis and progression from simple steatosis to metabolic dysfunction-associated steatohepatitis (MASH), cirrhosis, and hepatocellular carcinoma. Bidirectional communication through the gut–liver axis further amplifies disease progression.

**Table 2:** Summary of host–microbiome–hormonal interactions and mechanistic pathways in MASLD pathogenesis and therapy.

Pathway/mechanism	Key mechanism	Pathogenic consequence	Clinical/therapeutic implication
Gut Microbiota & Inflammatory Signaling	Dysbiosis → ↑ IL1R1 signaling via IL-1α/β → MyD88 & IRAK → NF-κB & MAPK activation (18)	Hepatocyte stress, ballooning, fibrosis, MASH progression; altered bile acid–FXR/TGR5 signaling (18)	IL1R1 blockade may reduce microbiota-driven hepatic inflammation (18)
Host Genetics & the Microbiome	PNPLA3, TM6SF2, MBOAT7, HSD17B13, GCKR variants influence lipid metabolism & inflammation; genetic variants shape microbiota composition (19)	Dysbiosis (↑ pathobionts, ↓ SCFA producers); epigenetic remodeling via microbial metabolites; heritability of MASLD risk (19)	Genetic profiling could guide microbiome-targeted therapy; twin studies highlight host–microbiota heritability (19)
Epigenetic Modulation by Microbial Metabolites	SCFAs (e.g., butyrate) act as HDAC inhibitors → transcription of anti-inflammatory/oxidative genes; dysbiosis ↓ SCFAs, ↑ endotoxin/BCAAs (19)	Epigenetic reprogramming of hepatocytes; inflammation, fibrosis, HCC predisposition (19)	Restoring SCFA balance may prevent long-term hepatocarcinogenesis (19)
Thyroid Hormones in MASLD	Hypothyroidism → TSH-mediated insulin resistance, lipid dysregulation, ↓ FA oxidation; TSH alters microbiota (20)	MASLD progression, fibrosis risk ↑ with higher TSH (20)	THR-β agonists show anti-steatotic effects; thyroid hormone replacement may reduce MASLD risk (20)
Microbiota-Dependent Efficacy of THR-β Agonists	HSK31679 efficacy depends on microbiota (esp. *Bacteroides thetaiotaomicron*); produces sphingolipids → ↓ DC/macrophage activation (21)	Enhanced anti-inflammatory, anti-steatotic effects in microbiota-competent models (21)	Pharmacotherapy may need microbiome co-optimization; microbial enzymes = biomarkers & therapeutic targets (21)

↑, increased or upregulated; ↓, decreased or downregulated; →, leads to, promotes, or results in the subsequent effect/pathway.

### 3.1 Gut microbiota and inflammatory signaling in MASLD

The gut microbiota serves as a potent modulator of hepatic homeostasis. However, a key mechanistic pathway in microbiota-induced hepatic inflammation is regulated by interleukin-1 receptor type 1 (IL1R1). IL-1α/β will activate IL1R1 in order to recruit adaptor proteins such as myeloid differentiation primary response protein 88 (MyD88) and interleukin-1 receptor associated kinase (IRAK), therefore activating NF-kB and mitogen- activated protein kinases (MAPK) pathways, enhancing cytokine production and immune cell engagement, contributing to hepatocyte stress (18).

IL1R1 signaling can also be amplified by the alteration in gut microbiota (dysbiosis), leading to increase in intestinal permeability and translocation of microbial products, triggering IL1R1 signaling and downstream inflammation. This process will lead to ballooning degeneration, fibrosis and progression towards MASH. Lipid and glucose metabolism are altered by IL1R1 interaction with gut microbiota-regulated bile acid signaling through FXR and the TGR5 pathways (18).

### 3.2 Host genetics and the microbiome: a bidirectional relationship

Genome-wide association studies have identified multiple MASLD-susceptibility loci such as PNPLA3, MBOAT7, HSD17B13, TM6SF2 and GCKR, which can alter hepatic lipoprotein secretion, inflammatory signaling and lipid metabolism (19). The genetic modifications can also have an impact on the gut microbiota’s function and composition. For instance, individuals harboring PNPLA3 risk alleles exhibit a dysbiotic gut community, marked by an overabundance of pathobionts and a loss of SCFAs-producing commensals, while, in turn, microbiota-derived metabolites feedback to reshape hepatic gene expression through epigenetic mechanisms, thereby driving MASLD progression. Twin studies corroborate this idea by demonstrating that monozygotic twins exhibit a more analogous microbiota compared to dizygotic twins, indicating a heritable aspect of microbial ecology that relates to MASLD risk (19).

### 3.3 Epigenetic modulation by microbial metabolites

Hepatocyte gene expression is affected by microbial metabolites, through DNA methylation, histone alteration and regulation of non-coding RNAs. For example, butyrate, which is a microbial SCFA, acts as a histone deacetylase inhibitor, therefore promoting transcription of genes that are implicated in anti-inflammatory signaling and oxidative metabolism (19).

Dysbiosis leads to reduced SCFA production and increased harmful components such as branched-chain amino acids and endotoxin, further damaging hepatocytes. These long-term transcriptional changes will predispose individuals to HCC (19).

### 3.4 Thyroid hormones in MASLD: a hormonal–microbial–metabolic axis

Thyroid hormones are crucial modulators of hepatic mitochondrial function, lipogenesis and cholesterol metabolism. More specifically, overt hypothyroidism and subclinical hypothyroidism have been consistently associated to MASLD through thyroid stimulating hormone (TSH)-medicated hepatic insulin resistance, lipid dysregulation and reduced fatty acid oxidation (20).

Mendelian randomization studies, along with large-scale observational cohorts that came across a dose-dependent relationship between elevated TSH and advanced fibrosis in MASLD. Moreover, thyroid hormones were found to be implicated in the alteration of gut microbial composition, emphasizing that hypothyroidism has a dual effect on the pathogenesis of MASLD, both directly via hepatic metabolism modification and indirectly via gut microbiota modification (20). This has led to many trials of thyroid hormone receptor-Betta (THR-β) selective agonists demonstrating lipid-lowering as well as anti-steatotic effects, more prominently in patients with subclinical hypothyroidism and dyslipidemia (20).

### 3.5 Microbiota-dependent efficacy of THR-β agonists

HSK31679, which is a THR-β agonist, demonstrates how gut microbiota alters drug efficacy in MASLD. In fact, several comparative murine studies showed that the latter outperformed MGL-3196 in improving steatohepatitis, however, only in specific pathogen free mice, not germ free models, suggesting a microbiota-dependent mechanism (21). In addition, if HSK31679 is enriched with Bacteroides thetaiotaomicron, it modulates hepatic immunity by reducing dendritic cell and macrophage activation, therefore attenuating hepatic inflammation, by glucosylceramide synthase-derived sphingolipids production from the Bacteroides species (21). These results underscore a pharmacomicrobiomic paradigm in which gut microbial enzymes modulate metabolic drug efficacy, establishing microbial glucocorticoids as both a biomarker and a prospective therapeutic target in MASLD (21).

To summarize, understanding the crosstalk between host genetics, microbiota, hormones, and immune signaling offers several clinical opportunities. First, targeting IL1R1 may benefit MASLD patients with microbiota-driven inflammation.

Second, THR-β agonists such as HSK31679 may require co-optimization of the gut microbiome for maximal efficacy. Third, thyroid hormone replacement in subclinical hypothyroidism should be considered in MASLD risk stratification and finally, host genetic and epigenetic profiling may guide microbiome- or metabolite-targeted interventions. MASLD is not merely a hepatic condition but a system-level disorder involving metabolic, genetic, microbial, and endocrine networks. The gut microbiota is both shaped by and a shape of host susceptibility, immune signaling, and therapeutic response. A holistic model integrating host genetics, epigenetic reprogramming, hormonal axes, and microbiota-derived cues is essential for precision hepatology. Future MASLD therapies should be microbiome-aware, biomarker-guided, and genetically stratified to maximize efficacy and reduce progression.

## 4 Microbiota composition & disease correlation

### 4.1 Fecal, duodenal, and tissue microbiota signals in MASLD

Multiple studies have demonstrated a link between MASLD and fecal composition, in fact, alterations in the gut microbiome and fecal metabolites are commonly associated with MASLD. However, taxa vary by geography and methods. For instance, MASLD is, on one hand, associated with decreased *Faecalibacterium* and *Bifidobacterium*, but on the other hand higher *Dorea*, *Blautia*, and *Ruminococcus*. Thus, the disease is characterized by a shift in the microbiome marked by reduced abundance of well-known SCFA-producing taxa and enrichment of genera with potential pro-inflammatory roles. Mechanistic studies consistently report a depletion of SCFA-producing commensals and an enrichment of Gram-negative pathobionts capable of producing LPS and other pro-inflammatory mediators, thereby linking microbial community shifts to inflammatory processes (22).

A cross-sectional endoscopic study has demonstrated that duodenal mucosal microbiota exhibit disease-related changes in patients with MASLD, as it is anatomically close to the liver. The main in the duodenal mucosal microbiota was closely linked to liver fat content and stiffness seen on magnetic resonance imaging (MRI). In fact, gut microbes near the liver may help identify the degree of how much advanced the liver disease is, with an 81% accuracy (23).

Researchers were also looking for microbial signals in the liver and fat tissues. A low-biomass study found different bacterial DNA between two tissues across MASLD stages. For instance, *Enterococcus*, *Granulicatella*, and *Morganellaceae* were associated with MASH, whereas Brevibacterium were associated with fibrosis. This pattern indicates that there is a possible movement of bacteria into tissues, or the presence of tissue-specific microbial profiles (17).

### 4.2 Small-intestinal bacterial overgrowth and proximal dysbiosis

Although microbial composition is a remarkable factor in disease progression, excessive small-intestinal bacterial growth may indicate increased risk. In fact, a higher prevalence of fatty liver was found in individuals with SIBO. This underlines that a gut problem may increase harmful toxins release, therefore disturbing bile acid balance in MASLD (24).

### 4.3 Diet, microbiota diversity, and MASLD risk

Diet-related gut microbial diversity is linked to a decreased risk of MASLD. A score based on foods associated with increased microbial diversity and short-chain fatty acid production demonstrated that an increase in this score corresponded to a reduction in MASLD’s odds by approximately 10%. This score is the Dietary Index of Gut Microbiota (DI-GM). Mediation analyses showed that the body mass index (BMI) and inflammation index like C-reactive protein (CRP) played a major role in this association (59% and 16% respectively) (25). This finding supports the idea that dietary approaches aimed at increasing microbial diversity, therefore altering gut microbiota, in turn affecting hepatic metabolism and inflammation, leading to a reduction in MASLD risk (25).

### 4.4 Microbial metabolites and host pathways

Changes in gut biochemistry, influenced by microbial affected pathways, reflect and may contribute to liver disease characteristics. In fact, gut microbiota metabolites, as well as amino acids, fatty acids and acylcarinitines are key metabolic markers of MASLD (26). For example, LPS movement will trigger TLR4 signaling, eventually increasing glutaminase-1 levels leading to high ammonia. This mechanism underlines the link between excess Gram negative bacteria and enhanced metabolic stress and hepatic inflammation (22, 26). In addition, interaction between bile acids and microbiota through FXR/TGR5 receptors, as well as reduction in SCFA producing bacteria demonstrates the link between microbial composition and liver fat metabolism and inflammation (26).

### 4.5 Correlates of disease severity and clinical phenotyping

As previously mentioned, mucosal and tissue-based studies have further strengthened the link between microbiota and MASLD, further supporting the idea that microbial signals vary in line with the severity seen on histology (17, 23). Moreover, non-invasive fibrosis score tools are crucial for assessment of disease severity across study groups and for linking paternal microbial organisms to established clinical outcomes (27).

### 4.6 Extra-hepatic integration and the bone–liver–gut triad

Since MASLD is closely linked to systemic metabolic networks, any change in its composition may have extra-hepatic consequences. Compromised intestinal barrier function, gut dysbiosis and inflammatory mediators are tied to bone turnover and mineral metabolism. Thus MASLD-associated dysbiosis may impact organs, other than the liver, via common inflammatory mechanisms and SCFA-related pathways (28).

### 4.7 Limitations of the current evidence

Three recurring methodological challenges are evident in this body of research. First, differences between fecal, mucosal and tissue samples present different microbial milieu, therefore their results are not directly comparable, and microbial profiles from proximal sites (for example the duodenum) may have stronger associations with hepatic outcomes than stool samples alone (23). Second, strict contamination control should be done in studies involving low-biomass tissues, because when such measures are employed, detectable microbial signals may often persist. Those signals must be interpreted cautiously and confirmed using independent validation methods (17). Finally, variability in populations such as medication use, diet differences and metabolic comorbidities can make it more challenging to detect consistent patterns in microbiome composition across individuals or groups. Therefore, standardized characteristics, such as certain BMI ranges, should be incorporated in order to be able to classify the direct versus indirect effects of the microbiota (22, 25).

### 4.8 Implications and future directions

MASLD is associated with a decreased beneficial SCFA-producing bacteria and an increase in potentially harmful pathobionts, especially in areas close to the liver (22). These microbial compositions alterations are essential metabolic features (microbe-derived inflammatory molecules, altered bile acids and SCFAs) (22). Microbial signals from both tissues and mucosal compartments are associated with disease presence and severity. In addition, a diversity index based on dietary intake shows an inverse correlation with MASLD and appears to influence the disease through its effect on systemic inflammation and body fat, shedding lights on potential targets for intervention (25). Finally, from a clinical point of view, combining reliable non-invasive staging methods with mucosal or tissue microbiome profiling could enhance risk assessment and support clinical trials aimed at dictating whether a specific diet or microbiota-based interventions can directly improve hepatic outcomes (25–28).

In summary, MASLD is consistently linked to changes near the liver, and sometimes in liver tissue itself. Those changes are connected to metabolites and inflammation, likely driving liver damage. Lower MASLD risk is tied to diet and greater microbial diversity, partly through effects of BMI and inflammation, while microbiomes targeting mucosal surfaces relate to fibrosis extent and severity. Finally, clinically focused and well-designed studies that account for sample type are key to turning these associated into actionable treatments.

## 5 Microbiome-targeted therapeutics in MASLD

Recent advances highlight the gut–liver axis as a key modulator of MASLD. Accordingly, multiple microbiome-focused therapies have been explored to restore intestinal homeostasis and ameliorate hepatic steatosis, inflammation, and fibrosis (**Table 3**). These approaches include supplementation with beneficial microbes or substrates (probiotics, prebiotics, synbiotics), delivery of microbial metabolites or components (postbiotics), transplantation of whole microbial communities (fecal microbiota transplantation (FMT)), as well as emerging bioengineered strategies (engineered bacteria and bacteriophages) (**Figure 2**). Each modality aims to counteract dysbiosis and its downstream effects on the liver through distinct mechanisms, as discussed below.

**Table 3:** Summary of microbiome-targeted therapeutics in MASLD.

Therapeutic approach	Proposed mechanisms	Evidence/findings	References
Probiotics	• Modulate gut microbiota (↑ Lactobacillus, Bifidobacterium) • Strengthen gut barrier (tight junctions) • Reduce endotoxemia and hepatic inflammation • Produce SCFAs and antimicrobial peptides	Preclinical studies: reduced liver fat & injury. Clinical: improved liver enzymes, glucose & lipids. Mixed results on steatosis/fibrosis progression.	(29–30)
Prebiotics	• Fermentable fibers → SCFA production (butyrate, propionate, acetate) • Enhance gut barrier & insulin sensitivity • Modulate bile acids (FXR–FGF19 axis) • Reduce hepatic lipogenesis & inflammation	Animal studies: ↓ liver TG & cytokines. Human trials: modest improvement in insulin resistance, aminotransferases, and lipids. Inconsistent impact on steatosis/fibrosis.	(29, 31)
Synbiotics	• Combine probiotics + prebiotics → synergistic effects • Enhance colonization & activity of probiotics • Improve gut barrier, endotoxin reduction, bile acid metabolism	Meta-analysis: ↓ hepatic steatosis, TNF-α, liver enzymes. Improved HDL and lipid profile. One trial: no significant effect on MRI steatosis/fibrosis.	(32–33)
Postbiotics	• Deliver microbial metabolites (SCFAs, tryptophan metabolites) • Strengthen gut barrier & reduce NF-κB signaling • Anti-inflammatory & antioxidant effects	Animal & early human studies: pasteurized Akkermansia muciniphila improved insulin sensitivity, reduced inflammation. No MASLD-specific trials yet.	(31, 34)
FMT	• Restore microbial diversity & suppress endotoxin-producing bacteria • Improve gut barrier function • Activate bile acid–FXR–FGF19 signaling • Introduce beneficial microbes & metabolites	Preclinical: transfer of MASLD phenotype via microbiota. Clinical: improved gut permeability, microbiome diversity, ↓ liver steatosis in some trials. Well-tolerated but variable efficacy.	(31, 35–36)
Engineered Bacteria	• Genetically modified strains deliver cytokines, peptides (IL-22, GLP-1) • Improve gut barrier, insulin sensitivity, and reduce inflammation	Animal models: engineered Lactobacillus reduced steatohepatitis, improved metabolic outcomes. No MASLD-specific clinical trials yet.	(37–38)
Bacteriophage Therapy	• Target pathogenic gut bacteria (e.g., alcohol-producing Klebsiella) • Reduce LPS/endotoxin burden • Precision modulation of microbiota	Animal studies: phages reduced Klebsiella burden, improved liver inflammation & steatosis. No MASLD human trials yet.	(39–41)

↑, increased or upregulated; ↓, decreased or downregulated; →, leads to, promotes, or results in the subsequent effect/pathway.

**Figure 2:**
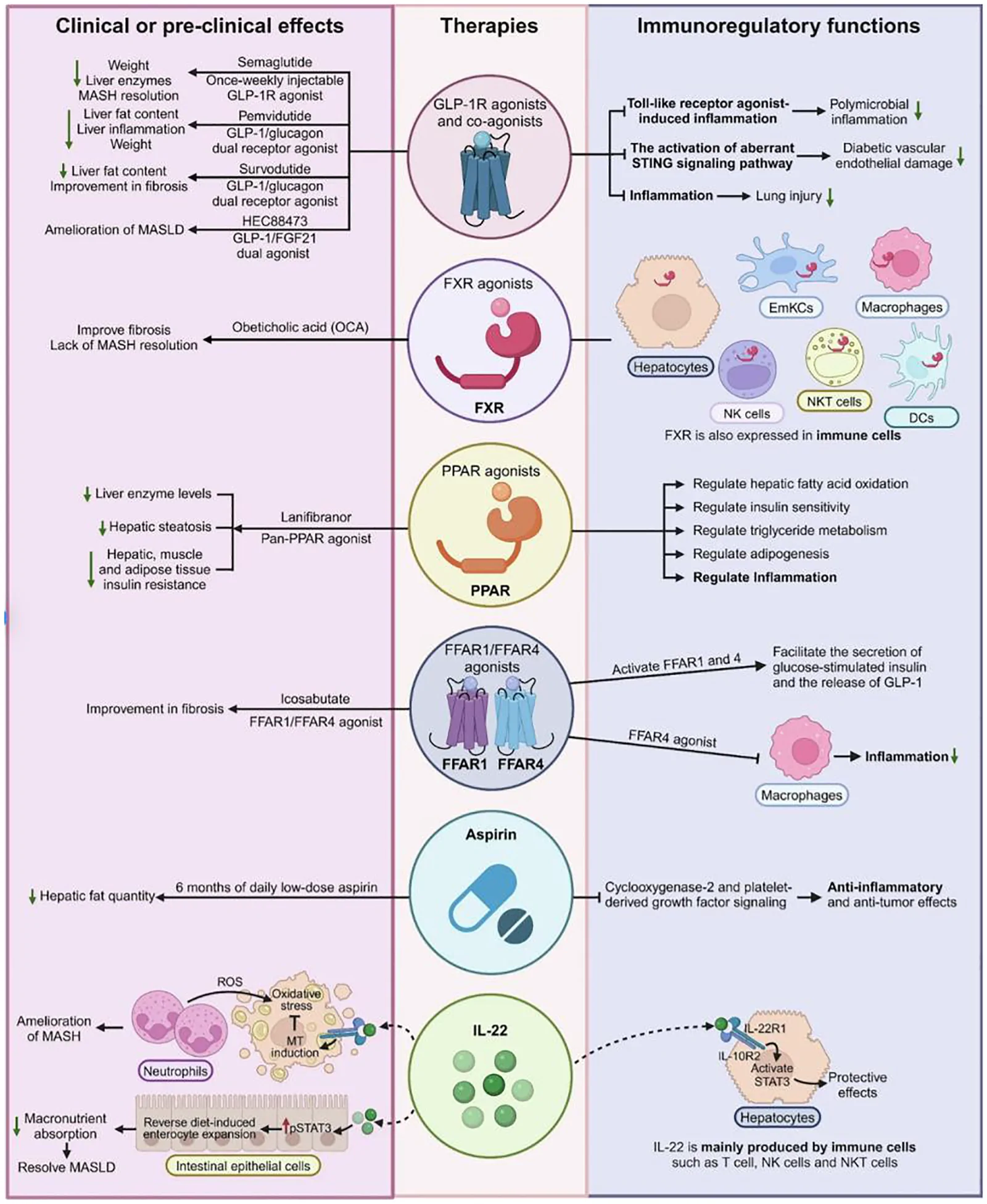
The different therapeutic approaches for the treatment of MASLD. This image summarizes interventions targeting gut microbial composition and function, including dietary modification, probiotics, prebiotics, synbiotics, postbiotics, fecal microbiota transplantation (FMT), engineered bacteria, and bacteriophage therapy. These approaches aim to restore microbial diversity, improve intestinal barrier integrity, reduce endotoxemia and systemic inflammation, modulate bile acid and short-chain fatty acid metabolism, and ultimately attenuate hepatic steatosis, inflammation, and fibrosis. Established interventions with clinical evidence are distinguished from emerging therapies that remain primarily at the preclinical or early clinical development stage.

### 5.1 Probiotics

Evidence supporting probiotics is derived from both experimental models and randomized clinical trials; however, clinical studies have generally demonstrated modest and heterogeneous benefits compared with the consistently favorable findings observed in animal models.

Probiotics are live microorganisms that confer health benefits to the host and have been tested as a therapy for MASLD. In the context of MASLD, probiotics (commonly strains of *Lactobacillus* and *Bifidobacterium*) can favorably modulate gut microbiota composition and function. Proposed mechanisms include enhancement of the intestinal barrier (upregulating tight junction proteins like zonulin and claudins to reduce gut permeability) and suppression of endotoxemia and inflammation. Probiotic organisms also produce metabolites such as SCFAs and antimicrobial peptides that may improve hepatic and systemic metabolism (for example, by reducing luminal LPS and down-regulating inflammatory signaling in the liver). Through these actions, probiotics have the potential to interrupt key pathogenic “hits” in MASLD, including microbial antigen translocation and chronic hepatic inflammation (29).

Evidence from numerous preclinical studies supports probiotic benefits in reducing liver fat accumulation and injury. However, human trials have yielded mixed results. Early meta-analyses suggested that probiotics may improve liver enzymes, liver fat, and even liver stiffness measurements in MASLD. There is substantial evidence for probiotic-induced reductions in alanine and aspartate aminotransferase levels (reflecting lower hepatocellular inflammation (29). Improvements in glucose homeostasis and blood lipids have also been reported in some trials, likely via enhanced insulin sensitivity and lipid metabolism. Despite these favorable trends, not all studies have seen consistent effects on hepatic steatosis or fibrosis progression (29). Heterogeneity in probiotic strains, dosing, and treatment duration may underlie the variable outcomes (31). Notably, a recent systematic review and meta-analysis found that probiotic supplements alone did not significantly improve key clinical endpoints in MASLD patients (30). This underscores that while probiotics are safe and biologically plausible, they might require optimization or combination with other interventions to achieve meaningful clinical impact in MASLD.

### 5.2 Prebiotics

Most mechanistic evidence for prebiotics originates from animal studies, whereas available human trials demonstrate improvements primarily in metabolic biomarkers rather than consistent histological liver outcomes.

Prebiotics are fermentable dietary fibers or other indigestible compounds that selectively stimulate the growth or activity of beneficial gut bacteria. By serving as substrates for commensal bacteria, prebiotics increase the production of SCFAs (such as butyrate, propionate, and acetate) and other microbial metabolites that support intestinal and metabolic health. In MASLD, enhanced SCFA production is thought to strengthen the gut barrier (butyrate is a key energy source for colonocytes, promoting tight junction integrity) and to modulate host energy balance and inflammation. SCFAs can engage receptors (e.g., GPR41/43) and enteroendocrine pathways to improve insulin sensitivity, increase satiety hormones, and reduce hepatic lipogenesis. Prebiotic-driven shifts in microbiota can also alter bile acid profiles toward more secondary bile acids, which activate the ileal FXR and fibroblast growth factor-19 (FGF19) endocrine axis to suppress hepatic fat synthesis. Collectively, these mechanisms illustrate how prebiotics favorably tweak the gut–liver metabolic crosstalk (31).

Animal models of MASLD (and related metabolic syndrome) have demonstrated that adding fermentable fibers can reduce liver TG accumulation and inflammatory cytokine levels, in parallel with enrichment of SCFA-producing taxa and upregulation of tight junction proteins in the intestine (31). Human data, while somewhat limited, suggest modest metabolic benefits from prebiotics. Some randomized trials in non-alcoholic fatty liver disease reported improved insulin resistance and reductions in body weight or aminotransferases with prebiotic supplementation (e.g., inulin or fructooligosaccharides). Meta-analyses indicate that prebiotic and fiber interventions can significantly lower fasting insulin levels and homeostatic model assessment (HOMA-IR), reflecting better glycemic control. Beneficial changes in lipid profiles (lower TGs, total cholesterol, and LDL) have also been observed. However, similar to probiotics, the impact of prebiotics on hepatic steatosis or fibrosis in MASLD patients is inconsistent across studies (29). Many trials are short-term and use surrogate endpoints; thus, while prebiotics are a promising adjunct to diet therapy, more robust clinical data are needed to confirm their long-term hepatic benefits in MASLD.

### 5.3 Synbiotics

Among microbiome-directed nutritional interventions, synbiotics currently possess the strongest clinical evidence, supported by several randomized controlled trials and meta-analyses demonstrating modest improvements in steatosis and metabolic parameters.

Synbiotics combine probiotics and prebiotics in a single formulation, aiming for synergistic effects on the gut ecosystem. The prebiotic component enhances survival and activity of the co-administered probiotic strains in the colon, potentially amplifying their microbiome-modulating impact. In MASLD, synbiotics have shown promise compared to probiotics or prebiotics alone. By jointly delivering beneficial microbes and the fibers they ferment, synbiotics can more effectively increase the abundance of anti-inflammatory commensals (such as *Lactobacillus*, *Bifidobacterium*, or SCFA-producing anaerobes) while suppressing pathobionts linked to liver disease. Mechanistically, synbiotics are thought to exert the combined actions described above: reinforcement of gut barrier function, reduction of endotoxins, modulation of bile acid metabolism, and systemic metabolic improvements (lipids and insulin sensitivity) (32).

Clinical evidence suggests that synbiotics may yield tangible improvements in MASLD outcomes. A recent meta-analysis of randomized trials found that synbiotic therapy led to significant reductions in liver fat content and inflammatory markers in MASLD patients. In that analysis, synbiotics (often comprising mixtures of *Lactobacillus*/*Bifidobacterium* with inulin or fructooligosaccharides) decreased hepatic steatosis and TNF-α levels, and improved the lipid profile (notably raising high-density lipoprotein (HDL) cholesterol) (42). Importantly, liver enzyme elevations alanine aminotransferase and aspartate aminotransferase tended to improve as well, suggesting a reduction in hepatocellular injury (42). These benefits were generally observed over intervention periods of around 12 weeks, which appears to be an adequate duration to detect changes. Other reviews corroborate that combined pre/probiotic therapy can positively influence metabolic parameters (insulin resistance, serum lipids) in MASLD. Still, not all trials have been uniformly positive – for example, one year-long synbiotic trial did not significantly change MRI-measured steatosis or fibrosis, possibly due to patient or regimen differences (33). Overall, synbiotics represent a promising approach, and the consistency of their beneficial effects in meta-analyses indicates that restoring gut eubiosis with a multifaceted strategy can translate into improved liver health.

### 5.4 Postbiotics

Current evidence for postbiotics remains largely preclinical, with only limited early-phase human studies evaluating metabolic outcomes and no definitive clinical evidence specifically in MASLD.

Postbiotics are bioactive products of microbes – these include non-viable microbial cells, cellular components, or metabolites that can confer health benefits without requiring live bacterial colonization (34). Examples of postbiotics range from bacterial fermentation products (SCFAs, tryptophan metabolites), to purified cell wall components (peptidoglycans, lipoteichoic acids), to heat-killed beneficial bacteria preparations (31). The appeal of postbiotics lies in harnessing the beneficial molecules produced by the microbiome while avoiding potential risks of live organism administration. In MASLD, postbiotics could favorably modulate the gut–liver axis by delivering key microbial metabolites that reduce inflammation, improve insulin sensitivity, and protect the intestinal barrier. For instance, butyrate and other SCFAs administered directly have been shown to strengthen gut barrier integrity and exert anti-inflammatory effects in the liver via inhibition of NF-κB signaling and promotion of regulatory immune responses (31). Likewise, bacterially derived antioxidants or bile-acid derivatives could mitigate oxidative stress and fibrogenic signaling in the liver.

Though clinical research on postbiotics in MASLD is still nascent, early findings are encouraging. A notable example is *Akkermansia muciniphila*, a mucin-degrading commensal often depleted in metabolic disorders. A pasteurized (heat-killed) preparation of *A. muciniphila* – considered a postbiotic since the bacteria are not alive – was shown to improve metabolic parameters in obese and insulin-resistant humans (31). In those studies, pasteurized *A. muciniphila* supplementation led to reductions in body fat, improved insulin sensitivity, and lowered inflammatory markers, without the safety concerns of live microbe therapy (31). These anti-obesity and anti-inflammatory effects suggest potential applicability to MASLD, wherein insulin resistance and chronic inflammation drive disease progression. Other postbiotic candidates include purified probiotic metabolites (e.g., bacteriocins or exopolysaccharides) that have been shown *in vitro* to inhibit pathogenic gut bacteria and reduce inflammatory responses (31). While no trials have yet reported postbiotic use specifically in MASLD patients, this approach holds promise. It could provide a targeted means to deliver the beneficial signaling molecules of a healthy microbiome (such as SCFAs or secondary bile acids) to the host, thereby improving hepatic outcomes without altering the microbiota composition per se. Further research is needed to identify the most effective postbiotic compounds and confirm their impacts on liver steatosis and fibrosis *in vivo*.

### 5.5 Fecal microbiota transplantation

FMT has demonstrated convincing mechanistic efficacy in animal models, whereas human studies remain limited to small pilot trials with encouraging but preliminary clinical findings.

FMT involves the transfer of a whole gut microbial community from a healthy donor into a recipient, with the goal of engrafting a more eubiotic microbiome. This strategy addresses MASLD’s dysbiosis at a holistic level, potentially reconstituting microbial diversity and functionality that confer metabolic benefits (35). Recent research has established multiple plausible mechanisms by which FMT might improve MASLD pathophysiology. Proposed effects include:

#### 5.5.1 Reduction of endotoxin-producing bacteria

Donor microbiota can replace or suppress Gram-negative pathobionts that generate LPS, thereby lowering portal endotoxemia and hepatic inflammation (43).

#### 5.5.2 Restoration of gut barrier integrity

FMT from healthy donors has been shown to “repair” the intestinal mucosal barrier, reducing gut permeability (leaky gut) through both mechanical (mucus, tight junctions) and immune-mediated mechanisms. This limits translocation of microbial products that incite liver injury (44).

#### 5.5.3 Immune and signaling modulation

Transplanted microbiota can down-regulate receptors for damage-associated molecular patterns (DAMPs) and up-regulate beneficial hepatic receptors, such as insulin receptors, improving insulin sensitivity. FMT has also been associated with activation of the bile acid–FXR–FGF19 signaling pathway, which reduces hepatic lipogenesis, and with increased production of microbial metabolites (like secondary bile acids) that exert hepatoprotective effects (31).

#### 5.5.4 Phageome and metabolite effects

Donor stool brings not only bacteria but also bacteriophages and fungi that can reshape the recipient’s microbiome ecology. Modulation of the gut virome (phageome) may selectively control overgrowth of harmful bacteria. Additionally, FMT can introduce microbes that generate beneficial metabolites (e.g., SCFAs, indoles) which reduce inflammation and fibrosis in the liver (31).

Preclinical studies provided proof-of-concept for FMT in metabolic liver disease. Transplanting gut flora from healthy mice into high-fat diet mice (and vice versa) demonstrated that the microbiome itself can transmit or protect from steatosis and metabolic dysfunction. Building on animal data, early clinical trials of FMT in MASLD have been conducted. Safety findings are reassuring: FMT (whether via colonoscopy, enema, or capsules) has been well-tolerated in MASLD patients, with no serious adverse events reported in pilot studies (35).

Efficacy signals, while preliminary, indicate that FMT can beneficially impact the liver and metabolism. In one randomized pilot trial, a single allogeneic FMT from a lean healthy donor improved small-intestinal permeability in obese MASLD patients, suggesting restoration of gut barrier function. However, this study did not find significant short-term changes in MRI-measured liver fat or insulin resistance compared to an autologous FMT control, possibly due to the small sample and timing of assessments (43). Another study by Witjes et al. used serial FMTs from lean vegan donors in patients with MASH. The treated group showed a trend toward lower hepatic inflammation (necroinflammatory histology scores), along with favorable shifts in plasma metabolites and liver gene expression related to inflammation and lipid metabolism. Though these changes did not reach statistical significance due to an underpowered sample, they align with the mechanistic expectations of FMT (44). A more recent controlled trial in China compared FMT plus lifestyle intervention to probiotic plus lifestyle in MASLD; the FMT group had significantly greater reduction in liver steatosis as measured by the FibroScan controlled attenuation parameter, as well as increased gut microbiota diversity and abundance (36). This suggests FMT may outperform conventional probiotic supplementation in modulating the microbiome and improving hepatic fat content when combined with diet therapy.

Overall, FMT holds considerable promise in MASLD by delivering a broad consortium of microbes that can recalibrate the gut–liver axis. The therapy appears to improve gut permeability and even histologic inflammation in some patients. Nonetheless, not all FMT recipients exhibit a sustained engraftment or metabolic benefit, highlighting the importance of donor selection and protocol optimization. Donor factors (metabolic fitness, diet such as high-fiber intake, presence of specific beneficial taxa), recipient baseline microbiome, FMT dose/frequency, and use of concurrent therapies (e.g., diet, obesity medications) likely influence outcomes. Indeed, an ongoing placebo-controlled trial is now testing continuous FMT therapy via capsules over 24 weeks in MASH, combined with adjunctive next-generation probiotics (e.g., *Anaerobutyricum soehngenii*, pasteurized *A. muciniphila*, *Bifidobacterium animalis*) and a prebiotic (fructooligosaccharide) to maximize engraftment and efficacy (31). The results of such studies are eagerly awaited to determine whether FMT can reproducibly reduce MASLD activity and fibrosis. For now, FMT remains an experimental but compelling approach that addresses the microbiome comprehensively in MASLD.

### 5.6 Engineered bacteria

Evidence for engineered microbial therapeutics remains almost entirely preclinical, with no completed large-scale clinical trials in MASLD to date.

Engineered bacteria represent a novel therapeutic strategy wherein microbial strains are genetically modified to perform specific therapeutic functions *in vivo* (37). Unlike conventional probiotics that exert generic beneficial effects, engineered microbes can be designed to deliver drugs, cytokines, or other bioactive molecules directly in the gut, providing a targeted intervention for MASLD. This approach takes advantage of the localization and self-replicating nature of bacteria to achieve sustained delivery at the site of gut–liver interaction, potentially with fewer systemic side effects (37). For example, an engineered probiotic might be programmed to secrete a peptide hormone that enhances insulin sensitivity or an anti-inflammatory cytokine that dampens gut and liver inflammation. By responding to environmental cues (such as specific dietary components or inducing agents), these microbial “Trojan horses” can release therapeutic payloads when and where needed in the intestine. In MASLD, where multiple pathways (metabolic, inflammatory, fibrotic) are dysregulated, engineered bacteria offer the exciting possibility of simultaneously correcting several defects through microbial delivery of therapeutic molecules.

Early experimental models have demonstrated the feasibility and efficacy of engineered bacteria in liver disease. A classic prototype was the engineering of *Lactococcus lactis* (a food-grade bacterium) to produce human cytokines IL-10 and IL-2, initially for inflammatory bowel disease (37). This concept has since been extended to liver conditions. In a proof-of-concept study, *Lactobacillus* strains were engineered to secrete IL-22, a cytokine with regenerative and barrier-protective functions in the gut. Administration of IL-22-producing *Lactobacillus reuteri* in a mouse model of alcohol-induced steatohepatitis led to reduced bacterial translocation across the gut and significantly attenuated liver injury, steatosis, and inflammation (37). This highlights how strengthening gut barrier and immune signaling via engineered microbes can protect the liver. Another compelling example is the use of engineered bacteria to deliver glucagon-like peptide-1 (GLP-1). GLP-1 agonism is known to improve metabolic outcomes in MASLD, but pharmacologic GLP-1 analogues can have side effects (45). Researchers created strains of *Escherichia coli* Nissle 1917 and *Lactobacillus gasseri* that continuously secreted GLP-1 in the gut; in animal models, these microbes induced weight loss, improved insulin levels and lipid profiles, lowered liver enzyme levels, and even improved hepatic histology (38). Such results underscore the potential for engineered probiotics to address key components of MASLD (adiposity, dyslipidemia, insulin resistance, and liver inflammation) in an integrated manner.

Engineered bacterial therapies are still in preclinical or early clinical development for metabolic liver disease. To date, no large clinical trials have been completed in MASLD using this approach, but the platform is advancing rapidly. The favorable safety profile of commensal probiotic strains (like *Lactobacillus* spp. or *E. coli* Nissle) provides a foundation, and these can be further modified with synthetic biology tools. Researchers are even exploring next-generation techniques such as using CRISPR/Cas systems to modify the patient’s indigenous gut microbes *in situ*. In the future, one could envision editing resident microbiota to produce anti-fibrotic or anti-inflammatory compounds on demand, without introducing foreign species. While these innovations are on the horizon, engineered bacteria therapies will require rigorous testing to ensure containment of modified organisms and consistent therapeutic outputs. If successful, they hold the promise of *in vivo* biotherapeutic factories that correct metabolic and inflammatory derangements in MASLD in a highly targeted fashion.

### 5.7 Bacteriophage therapy

Bacteriophage therapy remains at the proof-of-concept stage, with evidence currently limited to experimental animal models and no published randomized clinical trials in MASLD.

Bacteriophages (viruses that infect bacteria) are emerging as precision tools to reshape the gut microbiome by selectively eliminating unwanted bacterial species (39). Phage therapy in MASLD is predicated on the idea that certain gut bacteria actively contribute to liver pathology – for instance, by producing hepatotoxic metabolites or proinflammatory molecules – and that removing or reducing these bacteria will alleviate disease (39). Unlike broad-spectrum antibiotics, phages can target specific bacterial strains without disturbing the overall microbiota, making them a compelling strategy to correct dysbiosis-related triggers of MASLD.

One striking example involves *Klebsiella pneumoniae* strains that produce high levels of endogenous alcohol in the gut. Such strains have been identified in a subset of MASLD patients and can cause “auto-brewery” effects that injure the liver (39). In a recent study, researchers isolated a lytic phage specific to an alcohol-producing *Klebsiella* and administered it to mice colonized with this bacterium (39). Phage treatment dramatically reduced the *Klebsiella* burden and prevented the development of steatohepatitis in these mice (39). Treated animals showed improvements in liver function, lower expression of inflammatory cytokines and lipogenic genes in the liver, and less fatty infiltration, without off-target harm to other commensals (39).

Beyond this example, other preclinical work has explored phage therapy targeting different gut bacteria implicated in liver disease. For instance, phages against *Enterococcus faecalis* (a bacterium producing cytolysins that promote liver injury in alcohol-related liver disease) have been shown to reduce hepatic inflammation and fibrosis in animal models (40). It is conceivable that similar approaches could target LPS-producing *Proteobacteria* or other pathobionts in MASLD to decrease endotoxemia and hepatic inflammation (40). Bacteriophage therapy remains in experimental stages for metabolic liver disease, and as of now no human clinical trials in MASLD have been published (40). There are challenges to address, including phage delivery (encapsulation to survive gastric acid), potential immune responses to phages, and ensuring that targeted bacteria are indeed pathogenic drivers. Nonetheless, the gut virome is a critical component of the microbiome, and phage populations naturally differ in MASLD vs. healthy individuals (40). Modulating the phageome through therapeutic phages or even FMT (which introduces donor phages) could become a future avenue to fine-tune microbiota composition. In summary, bacteriophage therapy offers a highly specific microbiome intervention for MASLD – essentially acting as a living antibiotic – and early data indicate it can safely alleviate liver pathology in at least certain dysbiosis-driven models (39).

In conclusion, microbiome-targeted therapeutics for MASLD encompass a spectrum from dietary supplements to engineered organisms and viruses, all aiming to disrupt the gut–liver pathogenic loop. Probiotics, prebiotics, synbiotics, and postbiotics have shown the ability to modulate the intestinal milieu, improve metabolic biomarkers, and partially attenuate liver injury, especially in early disease stages. More comprehensive interventions like FMT can re-establish microbial diversity and have demonstrated improvements in gut barrier function and liver inflammation in preliminary trials. Cutting-edge approaches such as synthetic probiotics and bacteriophages are in development to target specific pathways with high precision, holding promise for future MASLD therapies. While current evidence is encouraging, the field is still evolving. Larger randomized studies are required to confirm efficacy, optimize treatment regimens (e.g., ideal strains, dosing, and duration), and ensure long-term safety for these microbiome-focused therapies. Ultimately, integrating microbiome-targeted therapeutics with existing lifestyle and pharmacologic interventions could form a multi-pronged strategy to halt or even reverse MASLD progression via restoration of a healthy gut–liver axis.

## 6 Diagnostic tools, biomarkers, and research trends

### 6.1 Gut microbiota and metabolic biomarkers in MASLD

The diagnostic criteria for MASLD differ slightly from those used for the earlier term, metabolic-associated fatty liver disease, which had generated debate. Under the new guidelines, diagnosis requires imaging or histological confirmation of liver steatosis, along with at least one of five key cardiometabolic risk factors. In addition, the concept of steatohepatitis remains important in the disease framework. It has been renamed metabolic dysfunction–associated steatohepatitis to reflect the updated nomenclature while still maintaining continuity with previous studies on NASH (32). This change is intended to preserve the relevance of existing clinical trial data and biomarker research, ensuring they remain applicable to patients now diagnosed under the MASLD terminology. Whether using the broader term steatotic liver disease (SLD) or the more specific MASLD, the revised classification aims to improve awareness and recognition of the disease. It also offers a more precise way to differentiate types of fatty liver disease based on their underlying causes. By addressing the historical issue of disease variability, this framework supports progress in therapeutic development and biomarker discovery (32).

The microbiome refers to the complete collection of genetic material from the bacteria residing within the human body. Around 15 years ago, scientists began to suspect that the gut microbiota—particularly the inflammatory and metabolic effects triggered by LPS from Gram-negative bacteria—might play a role in the development of MASLD (46).

Initially, this hypothesis was supported mainly by findings from animal studies. However, research into the human microbiome and its connection to MASLD began to gain momentum, especially following the sequencing of the human gut microbiome. It soon became evident that gut microbes influence host metabolism—not only by enhancing the efficiency of energy extraction from food but also by altering metabolites from the diet or host that, in turn, affect metabolic pathways (46).

Over the past decade or so, a growing body of knowledge has highlighted the importance of the gut microbiota in both human health and disease. During this period, researchers faced unique challenges in studying microbiota’s role in obesity and its associated conditions like MASLD. These challenges stemmed largely from technical constraints in accurately identifying both microbial species and the metabolic products they generate. The distinct gut microbiome profiles and their metabolites offer promise as noninvasive biomarkers for MASLD diagnosis and staging. Furthermore, modulating the gut microbiota—via probiotics, prebiotics, diet, or FMT is being explored as a therapeutic approach (32).

### 6.2 Global research trends on gut microbiota and MASH: bibliometric and scientometric analysis

This bibliometric and scientometric study provides a comprehensive analysis of global research trends at the intersection of gut microbiota and MASH, which was formerly known as NASH. The analysis shows a clear upward trajectory in scientific interest and publication volume since 2011, with the number of studies reaching a peak in 2021. This growing body of literature reflects the increasing recognition of the gut-liver axis as a critical pathway in the pathogenesis of MASH (47). Geographically, China has become the most productive country in terms of publication output, indicating a strong domestic investment in this research area. However, the United States leads in terms of total citations and international collaborations, positioning it as a key influencer in the field. Prominent institutions contributing to this research include Shanghai Jiao Tong University and the University of California system, both of which are recognized for their high-quality outputs and extensive collaborative networks. In terms of dissemination, journals such as *Nutrients*, *Scientific Reports*, and the *World Journal of Gastroenterology* were identified as the most active and impactful publishing platforms for related studies (47).

Thematically, the research hotspots identified through keyword co-occurrence and cluster analysis include mechanisms involving bile acids, inflammation, the gut-liver axis, and the role of microbial metabolites such as SCFAs. These components have been shown to influence metabolic processes, immune responses, and liver inflammation, all of which contribute to the progression of MASH. Moreover, recent trends suggest an increasing focus on the development of therapeutic strategies that aim to modulate gut microbiota. These include the use of probiotics, prebiotics, synbiotics, and fecal microbiota transplantation, which are being investigated for their potential to restore microbial balance and attenuate liver injury (26). Additionally, there is a growing incorporation of multi-omics approaches—such as metagenomics, transcriptomics, proteomics, and metabolomics—allowing for a more integrative understanding of host-microbiome interactions at a systems biology level (47). This comprehensive exploration of research patterns and emerging trends highlights the central role of the gut microbiota in MASH and sets the stage for future investigations aimed at identifying precise microbial targets and novel intervention strategies. The study underscores the importance of interdisciplinary collaboration and advanced technological integration to further unravel the complex mechanisms linking gut dysbiosis to liver disease.

## 7 Clinical trials, patient-centered studies, and future directions

### 7.1 Epidemiology, diagnosis, and clinical framework of MASLD

Although the epidemiology and pathophysiological basis of MASLD have been discussed previously, their clinical implications deserve particular emphasis. Contemporary management increasingly relies on early identification of patients at greatest risk for progressive fibrosis using non-invasive fibrosis scores, elastography, and emerging biomarker panels. These tools facilitate risk stratification and help identify individuals who may benefit from specialist referral, liver biopsy, or pharmacological intervention. In parallel, the recent approval of the thyroid hormone receptor-β agonist resmetirom for selected patients with non-cirrhotic MASH and moderate-to-advanced fibrosis represents an important milestone in the transition toward mechanism-based therapy. Nevertheless, lifestyle modification remains the cornerstone of treatment across the entire MASLD spectrum, while pharmacological therapy should be viewed as complementary and targeted to carefully selected patient populations. This evolving clinical framework highlights the movement from a “one-size-fits-all” approach toward individualized management guided by disease stage, metabolic risk profile, and emerging molecular biomarkers (48).

### 7.2 Transition from MASLD to MASH and HCC: mechanistic and clinical insights

Rather than representing distinct disease entities, MASLD, MASH, and hepatocellular carcinoma constitute a continuous biological spectrum driven by persistent metabolic injury, chronic inflammation, fibrogenesis, and immune dysregulation. As discussed in earlier sections, multiple molecular pathways—including gut microbial dysbiosis, oxidative stress, genetic susceptibility, and altered immune signaling—interact to determine disease progression. From a clinical perspective, identifying patients at greatest risk for progression remains a major priority, emphasizing the need for improved biomarkers and personalized surveillance strategies (49, 50).

As MASLD advances to MASH, the sustained inflammatory milieu facilitates the infiltration of monocytes, neutrophils, and lymphocytes, amplifying tissue damage. Hepatic stellate cells (HSCs) become activated into myofibroblasts, producing excessive extracellular matrix (ECM) components that disrupt normal liver architecture and lead to fibrosis. Concurrently, gut microbiota dysbiosis exacerbates liver injury by increasing intestinal permeability, enabling translocation of bacterial products such as LPS and microbial metabolites into the portal circulation. These signals activate TLR-mediated pathways in Kupffer cells and other immune populations, perpetuating hepatic inflammation and fibrogenesis (50).

In the transition to HCC, long-standing oxidative stress, chronic inflammation, and fibrotic remodeling converge to create a pro-tumorigenic microenvironment. Fibrosis alters vascular architecture, inducing hypoxia, which activates hypoxia-inducible factors (HIFs) and pro-angiogenic pathways (50). The immune landscape becomes increasingly immunosuppressive, dominated by regulatory T cells (Tregs), myeloid-derived suppressor cells (MDSCs), and tumor-associated macrophages (TAMs), which inhibit cytotoxic T cell and natural killer (NK) cell activity (50). Simultaneously, hepatocytes subjected to chronic injury accumulate genomic mutations and epigenetic changes that impair cell-cycle regulation, enhance proliferation, and confer resistance to apoptosis. Together, these factors establish a permissive environment for malignant transformation, tumor progression, and metastasis, marking the final stage in the MASLD–MASH–HCC continuum (50).

### 7.3 The relationship between pathogenesis and possible treatments for the MASLD-cirrhosis spectrum

The increasing understanding of MASLD pathogenesis has shifted therapeutic development from treating isolated metabolic abnormalities toward targeting interconnected pathogenic pathways. Current therapeutic strategies aim to simultaneously improve metabolic dysfunction, attenuate hepatic inflammation, reduce fibrogenesis, and restore gut–liver homeostasis. This systems-based approach reflects growing recognition that effective disease modification will likely require combination therapies tailored to individual patient phenotypes rather than single-target interventions. Consequently, integrating lifestyle modification with emerging pharmacological agents and microbiome-directed therapies may provide the greatest opportunity to delay progression to cirrhosis and its complications (51).

Emerging approaches also focus on metabolic pathway modulation (such as FXR and PPAR agonists), immune modulation, and mitochondrial protective agents. By understanding the interplay between the disease’s pathophysiology and its therapeutic targets, clinicians and researchers can design multi-pronged strategies to slow or prevent the transition from early-stage MASLD to advanced cirrhosis.

### 7.4 Trends in MASLD clinical trials: insights from ClinicalTrials.gov

This study analyzed interventional clinical trials on MASLD registered on ClinicalTrials.gov to assess research trends, characteristics, and gaps. A total of 317 trials were identified, with a noticeable increase in registrations after the term MASLD was introduced, reflecting growing research interest. Most trials were single-center (66.9%), randomized (74.1%), and involved parallel assignment (72.9%). The majority were in phase II (41.6%) or phase III (28.1%), focusing largely on pharmacological interventions, particularly novel agents targeting metabolic pathways, inflammation, and fibrosis (52). Non-pharmacological studies, such as lifestyle interventions, were less common. Geographic distribution showed the highest trial activity in North America (46.7%) and Europe (29.3%), with limited representation from other regions, indicating uneven global research participation. Many trials had relatively small sample sizes, and only a minority reported results, highlighting the need for improved data transparency and completion rates. Overall, the findings emphasize the growing emphasis on MASLD therapies, the dominance of drug-related research, and the necessity for broader, more diverse, and transparent clinical investigations (52).

These trends illustrate a progressive shift from conventional lifestyle-focused interventions toward precision medicine approaches integrating pharmacological therapies, biomarker-guided patient selection, and microbiome-targeted interventions. Future clinical trials should increasingly incorporate multi-omics profiling, standardized microbiome characterization, and long-term histological outcomes to better identify patient subgroups most likely to benefit from individualized therapeutic strategies.

### 7.5 Patient self-management in MASLD: determinants and impact on outcomes

Patients with MASLD generally show limited self-management capacity, influenced by marital status, smoking, education, and disease severity. In a study of 241 patients, the mean self-management score was 91.24 ± 16.98, with 55.6% classified as low, 41.9% moderate, and only 2.5% high. Patients scored highest in avoiding unhealthy behaviors and lowest in daily life management. Multivariate analysis revealed better self-management among married patients, non-smokers, those with higher education, and those with more severe fatty liver (53). During one-year follow-up, readmission rates were 18.25% in the low group, 7.48% in the moderate group, and 0% in the high group. Enhancing education, family support, and smoking cessation may strengthen self-management and improve MASLD outcomes (53).

## 8 Conclusion

MASLD has emerged as a major global health challenge, driven by complex interactions among metabolic dysfunction, genetic susceptibility, gut microbiota dysbiosis, and immune and hormonal pathways. Although the approval of resmetirom marks a major advance in pharmacological management of selected patients with MASH, substantial unmet clinical needs remain because many patients fall outside the current approved indication or continue to progress despite therapy. The recognition of MASLD as a systemic disorder underscores the need for integrated, multi-targeted strategies that go beyond liver-focused approaches. Future directions point toward microbiome-modulating therapies, precision medicine guided by genetic and biomarker profiling, and the development of novel agents such as GLP-1 receptor agonists, bile acid modulators, antifibrotics, and engineered microbiome-based interventions. Advances in omics technologies and non-invasive diagnostic tools also hold promise for improving disease stratification and monitoring. Together, these innovations may pave the way for personalized, mechanism-driven therapies capable of halting or reversing MASLD progression and improving long-term outcomes. Collectively, the available evidence supports a systems biology model of MASLD in which genetic susceptibility, metabolic dysfunction, gut microbial ecology, immune activation, and environmental exposures interact dynamically throughout disease progression rather than acting as isolated pathogenic mechanisms. Future research should prioritize longitudinal, multi-center studies integrating genomics, transcriptomics, metabolomics, and microbiome profiling with standardized clinical phenotyping to identify reproducible biomarkers and therapeutic targets. Establishing causal microbial signatures, validating predictive multi-omics models, and conducting biomarker-guided precision medicine trials will be essential for translating mechanistic discoveries into individualized therapies capable of preventing disease progression and improving long-term clinical outcomes.

## Glossary

MASLD: Metabolic dysfunction–associated steatotic liver disease
NAFLD: Non-alcoholic fatty liver disease
NAFL: Non-alcoholic fatty liver
SFL: Simple fatty liver
NASH: Non-alcoholic steatohepatitis
MASH: Metabolic dysfunction–associated steatohepatitis
HCC: Hepatocellular carcinoma
TG: Triglycerides
SCFA: Short-chain fatty acids
SIBO: Small-intestinal bacterial overgrowth
LPS: Lipopolysaccharide
TNF-α: Tumor necrosis factor alpha
IL-1β: Interleukin 1 beta
TLR4: Toll-like receptor 4
NF-κB: Nuclear factor kappa B
FXR: Farnesoid X receptor
TGR5: Takeda G protein–coupled receptor 5
IPA: Indole-3-propionic acid
IAA: Indole acetic acid
AHR: Aryl hydrocarbon receptor
PXR: Pregnane X receptor
VLDL: Very low-density lipoproteins
TMAO: Trimethylamine N-oxide
ROS: Reactive oxygen species
IL1R1: Interleukin-1 receptor type 1
MyD88: Myeloid differentiation primary response 88
IRAK: Interleukin-1 receptor-associated kinase
MAPK: Mitogen-activated protein kinases
TSH: Thyroid stimulating hormone
THR-β: Thyroid hormone receptor-beta
CAP: Controlled attenuation parameter
HSCs: Hepatic stellate cells
ECM: Extracellular matrix
DAMPs: Damage-associated molecular patterns
Tregs: Regulatory T cells
MDSCs: Myeloid-derived suppressor cells
TAMs: Tumor-associated macrophages
FMT: Fecal microbiota transplantation
GLP-1: Glucagon-like peptide-1

## References

[1] Scorletti E Carr RM . A new perspective on NAFLD: Focusing on lipid droplets. J Hepatol. (2022) 76:934–45. doi: 10.1016/j.jhep.2021.11.009 34793866

[2] Lazarus JV Mark HE Anstee QM Arab JP Batterham RL Castera L . Advancing the global public health agenda for NAFLD: a consensus statement. Nat Rev Gastroenterol Hepatol. (2022) 19:60–78. doi: 10.1038/s41575-021-00523-4 34707258

[3] Liu C Liu T Zhang Q Jia P Song M Zhang Q . New-onset age of nonalcoholic fatty liver disease and cancer risk. JAMA Netw Open. (2023) 6:e2335511. doi: 10.1001/jamanetworkopen.2023.35511 37747732 PMC10520743

[4] Li Y Yang P Ye J Xu Q Wu J Wang Y . Updated mechanisms of MASLD pathogenesis. Lipids Health Dis. (2024) 23:117. doi: 10.1186/s12944-024-02108-x 38649999 PMC11034170

[5] American Association for the Study of Liver Diseases Latin American Association for the Study of the Liver European Association for the Study of the Liver . A call for unity: The path towards a more precise and patient-centric nomenclature for NAFLD. Hepatology. (2023) 78:3–5. doi: 10.1097/HEP.0000000000000412 37192775

[6] Nassir F . NAFLD: Mechanisms, treatments, and biomarkers. Biomolecules. (2022) 12:824. doi: 10.3390/biom12060824 35740949 PMC9221336

[7] Harrison SA Bedossa P Guy CD Schattenberg JM Loomba R Taub R . A phase 3, randomized, controlled trial of resmetirom in NASH with liver fibrosis. N Engl J Med. (2024) 390:497–509. doi: 10.1056/NEJMoa2309000 38324483

[8] Schnabl B Damman CJ Carr RM . Metabolic dysfunction–associated steatotic liver disease and the gut microbiome: Pathogenic insights and therapeutic innovations. J Clin Invest. (2025) 135:e186423. doi: 10.1172/JCI186423 40166938 PMC11957707

[9] Bäckhed F Ley RE Sonnenburg JL Peterson DA Gordon JI . Host-bacterial mutualism in the human intestine. Science. (2005) 307:1915–20. doi: 10.1126/science.1104816 15790844

[10] Cebi M Yilmaz Y . Epithelial barrier hypothesis in the context of nutrition, microbial dysbiosis, and immune dysregulation in metabolic dysfunction-associated steatotic liver. Front Immunol. (2025) 16:1575770. doi: 10.3389/fimmu.2025.1575770 40438102 PMC12116361

[11] Benedé-Ubieto R Cubero FJ Nevzorova YA . Breaking the barriers: The role of gut homeostasis in metabolic-associated steatotic liver disease (MASLD). Gut Microbes. (2024) 16:2331460. doi: 10.1080/19490976.2024.2331460 38512763 PMC10962615

[12] Martin-Grau M Monleón D . The role of microbiota-related co-metabolites in MASLD progression: A narrative review. Curr Issues Mol Biol. (2024) 46:6377–89. doi: 10.3390/cimb46070381 39057023 PMC11276081

[13] Mignini I Galasso L Piccirilli G Calvez V Termite F Esposto G . Interplay of oxidative stress, gut microbiota, and nicotine in metabolic-associated steatotic liver disease (MASLD). Antioxidants. (2024) 13:1532. doi: 10.3390/antiox13121532 39765860 PMC11727446

[14] Loomba R Friedman SL Shulman GI . Mechanisms and disease consequences of nonalcoholic fatty liver disease. Cell. (2021) 184:2537–64. doi: 10.1016/j.cell.2021.04.015 33989548 PMC12168897

[15] Samuel VT Shulman GI . The pathogenesis of insulin resistance: Integrating signaling pathways and substrate flux. J Clin Invest. (2016) 126:12–22. doi: 10.1172/JCI77812 26727229 PMC4701542

[16] Effenberger M Grander C Hausmann B Enrich B Pjevac P Zoller H . Apelin and the gut microbiome: Potential interaction in human MASLD. Dig Liver Dis. (2024) 56:932–40. doi: 10.1016/j.dld.2023.11.023 38087672

[17] Schwenger KJP Copeland JK Ghorbani Y Chen L Comelli EM Guttman DS . Characterization of liver, adipose, and fecal microbiome in obese patients with MASLD: Links with disease severity and metabolic dysfunction parameters. Microbiome. (2025) 13:9. doi: 10.1186/s40168-024-02004-7 39810228 PMC11730849

[18] Ku J Hsu J Li Y Wu L . Interplay among IL1R1, gut microbiota, and bile acids in metabolic dysfunction‐associated steatotic liver disease: A comprehensive review. J Gastroenterol Hepatol. (2025) 40:33–40. doi: 10.1111/jgh.16750 39343617 PMC11771549

[19] Ha S Wong VWS Zhang X Yu J . Interplay between gut microbiome, host genetic and epigenetic modifications in MASLD and MASLD-related hepatocellular carcinoma. Gut. (2025) 74:141–52. doi: 10.1136/gutjnl-2024-332398 38950910 PMC11671994

[20] Pu S Zhao B Jiang Y Cui X . Hypothyroidism/subclinical hypothyroidism and metabolic dysfunction-associated steatotic liver disease: Advances in mechanism and treatment. Lipids Health Dis. (2025) 24:75. doi: 10.1186/s12944-025-02474-0 40016726 PMC11866868

[21] Zhang YH Xie R Dai CS Gao HW Zhou G Qi TT . Thyroid hormone receptor-beta agonist HSK31679 alleviates MASLD by modulating gut microbial sphingolipids. J Hepatol. (2025) 82:189–202. doi: 10.1016/j.jhep.2024.08.008 39181210

[22] Long Q Luo F Li B Li Z Guo Z Chen Z . Gut microbiota and metabolic biomarkers in metabolic dysfunction–associated steatotic liver disease. Hepatol Commun. (2024) 8. doi: 10.1097/HC9.0000000000000310 38407327 PMC10898672

[23] Ren M Pan H Zhou X Yu M Ji F . Alterations of the duodenal mucosal microbiome in patients with metabolic dysfunction-associated steatotic liver disease. Sci Rep. (2024) 14:9124. doi: 10.1038/s41598-024-59605-3 38643212 PMC11032335

[24] Wang Z Tan W Huang J Li Q Wang J Su H . Small intestinal bacterial overgrowth and metabolic dysfunction-associated steatotic liver disease. Front Nutr. (2024) 11:1502151. doi: 10.3389/fnut.2024.1502151 39742106 PMC11685094

[25] Pu Y Tan Z Wu Y Zeng S He H Zhang J . Association of gut microbiota dietary index with metabolic dysfunction-associated steatotic liver disease: The mediating roles of inflammation and body mass index. Front Nutr. (2025) 12:1573636. doi: 10.3389/fnut.2025.1573636 40453716 PMC12123378

[26] Beyoğlu D Popov YV Idle JR . Metabolomic hallmarks of obesity and metabolic dysfunction-associated steatotic liver disease. Int J Mol Sci. (2024) 25:12809. doi: 10.3390/ijms252312809 39684520 PMC11640922

[27] Basil B Myke-Mbata BK Eze OE Akubue AU . From adiposity to steatosis: Metabolic dysfunction-associated steatotic liver disease, a hepatic expression of metabolic syndrome – current insights and future directions. Clin Diabetes Endocrinol. (2024) 10:39. doi: 10.1186/s40842-024-00187-4 39617908 PMC11610122

[28] Zhang J Sun Z Xu L Wang Y Wang Y Dong B . Unraveling the link between metabolic dysfunction-associated steatotic liver disease and osteoporosis: A bridging function of gut microbiota. Front Endocrinol. (2025) 16:1543003. doi: 10.3389/fendo.2025.1543003 40235664 PMC11997446

[29] Mitrović M Dobrosavljević A Odanović O Knežević-Ivanovski T Kralj Đ Erceg S . The effects of synbiotics on the liver steatosis, inflammation, and gut microbiome of metabolic dysfunction-associated liver disease patients-randomized trial. Rom J Intern Med. (2024) 62:184–93. doi: 10.2478/rjim-2024-0004 38421902

[30] Sanyal AJ Newsome PN Kliers I Østergaard LH Long MT Kjær MS . Phase 3 trial of semaglutide in metabolic dysfunction–associated steatohepatitis. N Engl J Med. (2025) 392:2089–99. doi: 10.1056/NEJMoa2413258 40305708

[31] Zhou T Jin Z Jiang R Li W . Gut microbiota modulation by Traditional Chinese Medicine: A translational strategy for metabolic dysfunction-associated steatotic liver disease. Front Pharmacol. (2025) 16:1600439. doi: 10.3389/fphar.2025.1600439 40556760 PMC12185430

[32] Abdelhameed F Mustafa A Kite C Lagojda L Dallaway A Than NN . Gut microbiota and metabolic dysfunction-associated steatotic liver disease (MASLD): Emerging pathogenic mechanisms and therapeutic implications. Livers. (2025) 5:11. doi: 10.3390/livers501001130654563

[33] Scorletti E Afolabi PR Miles EA Smith DE Almehmadi A Alshathry A . Synbiotics alter fecal microbiomes, but not liver fat or fibrosis, in a randomized trial of patients with nonalcoholic fatty liver disease. Gastroenterology. (2020) 158:1597–1610.e7. doi: 10.1053/j.gastro.2020.01.031 31987796 PMC7613160

[34] Vazquez-Uribe R Hedin KA Licht TR Nieuwdorp M Sommer MOA . Advanced microbiome therapeutics as a novel modality for oral delivery of peptides to manage metabolic diseases. Trends Endocrinol Metab. (2025) 36:29–41. doi: 10.1016/j.tem.2024.04.021 38782649

[35] Wu J Chen X Qian J Li G . Clinical improvement effect of regulating gut microbiota on metabolic dysfunction-associated steatotic liver disease: Systematic review and meta-analysis of randomized controlled trials. Clin Res Hepatol Gastroenterol. (2024) 48:102397. doi: 10.1016/j.clinre.2024.102397 38879003

[36] Jarmakiewicz-Czaja S Sokal-Dembowska A Filip R . Effects of selected food additives on the gut microbiome and metabolic dysfunction-associated steatotic liver disease (MASLD). Med (Mex). (2025) 61:192. doi: 10.3390/medicina61020192 40005309 PMC11857189

[37] Dong L Lou W Xu C Wang J . Naringenin cationic lipid-modified nanoparticles mitigate MASLD progression by modulating lipid homeostasis and gut microbiota. J Nanobiotechnol. (2025) 23:168. doi: 10.1186/s12951-025-03228-x 40038718 PMC11881431

[38] Lok J Iannone V Gómez-Gallego C Vazquez-Uribe R Sommer MOA Kolehmainen M . “ Engineered Escherichia coli Nissle 1917 expressing IGF1 and FGF19 reduce liver fat accumulation and restore microbial equilibrium in a metabolic dysfunction-associated steatotic liver disease mice model. Proceedings. (2024) 91(1):402. doi: 10.3390/proceedings2023091402

[39] Gan L Feng Y Du B Fu H TianÇÃO Z Xue G . Bacteriophage targeting microbiota alleviates non-alcoholic fatty liver disease induced by high alcohol-producing Klebsiella pneumoniae. Nat Commun. (2023) 14:3215. doi: 10.1038/s41467-023-39028-w 37270557 PMC10239455

[40] Wong GLH Gane E Lok ASF . How to achieve functional cure of HBV: Stopping NUCs, adding interferon or new drug development? J Hepatol. (2022) 76:1249–62. doi: 10.1016/j.jhep.2021.11.024 35589248

[41] Yanagida A Corujo-Simon E Revell CK Sahu P Stirparo GG Aspalter IM . Cell surface fluctuations regulate early embryonic lineage sorting. Cell. (2022) 185:1258. doi: 10.1016/j.cell.2022.03.015 35364033 PMC8970287

[42] Liu L Li P Liu Y Zhang Y . Efficacy of probiotics and synbiotics in patients with nonalcoholic fatty liver disease: A meta-analysis. Dig Dis Sci. (2019) 64:3402–12. doi: 10.1007/s10620-019-05699-z 31203554

[43] Saeed H Díaz LA Gil-Gómez A Burton J Bajaj JS Romero-Gomez M . Microbiome-centered therapies for the management of metabolic dysfunction-associated steatotic liver disease. Clin Mol Hepatol. (2025) 31:S94–S111. doi: 10.3350/cmh.2024.0811 39604327 PMC11925441

[44] Grefhorst A de Groen P Wortelboer K Seegers JFML Gül IS . Randomised double-blind placebo-controlled trial protocol to evaluate the therapeutic efficacy of lyophilised faecal microbiota capsules amended with next-generation beneficial bacteria in individuals with metabolic dysfunction-associated steatohepatitis. BMJ Open. (2025) 15:e088290. doi: 10.1136/bmjopen-2024-088290 39788762 PMC11784342

[45] Moolla A Poolman T Othonos N Dong J Smith K Cornfield T . Randomised trial comparing weight loss through lifestyle and GLP-1 receptor agonist therapy in people with MASLD. JHEP Rep. (2025) 7:101363. doi: 10.1016/j.jhepr.2025.101363 40342635 PMC12060445

[46] Zhang R Yan Z Zhong H Luo R Liu W Xiong S . Gut microbial metabolites in MASLD: Implications of mitochondrial dysfunction in the pathogenesis and treatment. Hepatol Commun. (2024) 8. doi: 10.1097/HC9.0000000000000484 38967596 PMC11227362

[47] Li Y Zhou Y Wang L Lin X Mao M Yin S . Emerging trends and hotspots in the links between the gut microbiota and MAFLD from 2002 to 2021: A bibliometric analysis. Front Endocrinol. (2022) 13:990953. doi: 10.3389/fendo.2022.990953 36329894 PMC9624192

[48] Corrao S Calvo L Granà W Scibetta S Mirarchi L Amodeo S . Metabolic dysfunction-associated steatotic liver disease: A pathophysiology and clinical framework to face the present and the future. Nutr Metab Cardiovasc Dis. (2025) 35:103702. doi: 10.1016/j.numecd.2024.07.019 39358105

[49] Jee YM Lee JY Ryu T . Chronic inflammation and immune dysregulation in metabolic-dysfunction-associated steatotic liver disease progression: From steatosis to hepatocellular carcinoma. Biomedicines. (2025) 13:1260. doi: 10.3390/biomedicines13051260 40427086 PMC12109540

[50] Wu Q Yang Y Lin S Geller DA Yan Y . The microenvironment in the development of MASLD-MASH-HCC and associated therapeutic in MASH-HCC. Front Immunol. (2025) 16:1569915. doi: 10.3389/fimmu.2025.1569915 40370443 PMC12074932

[51] Kumar AR Nair B Kamath AJ Nath LR Calina D Sharifi-Rad J . Impact of gut microbiota on metabolic dysfunction-associated steatohepatitis and hepatocellular carcinoma: pathways, diagnostic opportunities and therapeutic advances. Eur J Med Res. (2024) 29:485. doi: 10.1186/s40001-024-02072-3 39367507 PMC11453073

[52] Du H Huang J Wang Y Wang C Wang Y Hou L . Analyzing MASLD interventional clinical trial registration based on the ClinicalTrials.gov database. BMC Gastroenterol. (2025) 25:148. doi: 10.1186/s12876-025-03732-2 40055604 PMC11887356

[53] Lin X Bao S Yu Y Huang H Shu M . Self-management in patients with metabolic dysfunction-associated steatotic liver disease: influencing factors and impact on readmission. J Health Popul Nutr. (2024) 43:134. doi: 10.1186/s41043-024-00618-z 39217400 PMC11366125

